# A comparative study of the efficacy of instrument-assisted soft tissue mobilization and massage techniques in patients with patellofemoral joint pain

**DOI:** 10.3389/fmed.2023.1305733

**Published:** 2023-11-13

**Authors:** Yang Liu, Yidan Wang

**Affiliations:** ^1^SchoolGraduate School of Wuhan Sports University, Wuhan Sports University, Wuhan, China; ^2^Faculty of Sports and Exercise Science, Universiti Malaya, Kuala Lumpur, Malaysia

**Keywords:** instrument-assisted soft tissue mobilization, manual therapy, patellofemoral pain syndrome therapy, physiotherapy, Tui-Na manipulation

## Abstract

**Purpose:**

The aim of this study was to compare the clinical efficacy of instrument-assisted soft tissue mobilization (IASTM) and manipulative therapy Tui-na techniques in the treatment of patients with patellofemoral joint pain syndrome, and to evaluate their impact on pain relief, functional improvement, and joint range of motion.

**Methods:**

In this study, 25 patients with patellofemoral pain syndrome were enrolled, comprising of an intervention group of 13 patients who received IASTM treatment and a control group of 12 patients who received Tui-na manipulation therapy. The treatment cycle lasted for 4 weeks, featuring two interventions per week. Before treatment, the visual analog pain scale (VAS) of the knee, Lysholm score of the knee, modified Thomas test (MTT), and maximum isometric strength of the extensor muscles of the lower limbs were measured and recorded for both groups. After the first and last treatments, the aforementioned indexes were reassessed, and the maximum isometric muscle strength of the lower extremity extensors was measured only after 4 weeks of treatment had been completed.

**Results:**

There was no significant difference in the basic information of the two intervention groups (*p* > 0. 05). After the first treatment and 4 weeks of treatment, the Lysholm score in both groups significantly improved (*p* < 0. 05), indicating that both interventions can improve the function of patients’ lower limbs. However, the Lysholm score in the IASTM group significantly increased compared with that of the massage group after 4 weeks of treatment, indicating that its improvement in functional performance is superior. Both groups showed significant improvement in knee joint pain after the first treatment and 4 weeks of treatment (*p* < 0. 05), with the IASTM group having a lower VAS score and better pain improvement after 4 weeks of treatment. The strength of the two intervention groups significantly increased after the maximum isometric muscle strength test of the lower limb extensor muscles before and after 4 weeks of treatment (*p* < 0. 05). After the MTT test, the extension angle, deviation angle, and hip abduction angle of the tested legs in the two intervention groups were significantly reduced (*p* < 0. 001), indicating an improvement in lower limb joint mobility.

**Conclusion:**

Instrument-assisted soft tissue mobilization treatment and Tui-na manipulation therapy significantly reduced pain, improved knee flexibility, and increased range of motion of the lower extremity in patients with PFPS. However, IASTM treatment significantly improved pain and function and sustained pain in the short to medium-term post-trial period.

**Clinical trial registration:**

www.isrctn.com, ISRCTN88098928

## Introduction

Patellofemoral Pain Syndrome (PFPS) is a common knee joint disorder characterized by pain and discomfort between the patella (kneecap) and the femur. Research indicates that the prevalence of PFPS in the general population is as high as 22.7% (1). Its symptoms include anterior knee pain during activities such as walking, running, and jumping, as well as pain when ascending or descending stairs. Prolonged periods of knee flexion and sedentary sitting can exacerbate pain, with abnormal patellar trajectory and increased local joint stress as key triggers for patellofemoral pain syndrome during human movement (2, 3). The causes of abnormal patellar trajectory are related to the disorder of the lower limb force line (4), which includes anatomical abnormalities of lower limb structures, decreased muscle strength in the lower limb, imbalance of muscle tension around the knee joint, and sports trauma (5–7). Additionally, Sinaei et al. (2, 8–11) research suggests that PFPS symptoms may be linked to the weakening of the biceps femoris muscle, delayed activation of the medial femoral muscle, and over-activation of the lateral femoral muscle in relation to the medial gage. The imbalance of muscle tone in the muscle tissues around the knee joint is a significant factor in inducing pain (12, 13). For this comparative study with a randomized double-blind controlled intervention design, the objective was to investigate the effectiveness of both IASTM treatment and conservative unarmed Chinese Tui-na manipulation therapy interventions, for improvement of the abnormal muscle tone in the lower limbs of patients with PFPS, restoration of the knee function, and reduction or elimination of pain.

Instrument-assisted soft tissue mobilization (IASTM) therapy is a therapeutic technique that involves external intervention on the body surface to loosen soft tissues using special therapeutic tools. This technique has been proven effective in improving pain and strength in the patient’s joint range of motion (5). As a non-invasive treatment method, it is commonly used for soft tissue injuries (such as skeletal muscle, ligament, and fascia) and post-operative recovery (14–16). In this study, the primary therapeutic tool used was the fascial knife, which allows for rapid location, examination, and removal of fascial adhesions during treatment. This effectively inhibits the occurrence of soft tissue fibrosis and muscle degeneration (17). The fascial knife is also commonly used to address various issues in different body parts, such as muscle recruitment, movement restriction, pain during exercise, motor control, and chronic inflammation (6, 18, 19). During the trial, pressure-sliding treatment was applied to the soft tissues around the knee joint to improve myofascial pain, abnormal tension, and other symptoms through myofascial release. Five different types of knives were used for manipulation and treatment: Type M—Big M Knife, Type A—Shark Knife, Type S—Hook Knife, Type C—Probe Sweep Knife, and Type B—Bat Knife. Each knife has its own specific shape and function (20). Furthermore, to address the characteristics of the PFPS condition, IASTM treatment focuses on deeper relaxation of the quadriceps muscle by relieving hypertonic muscles and tendons around the knee joint, thus improving elbow mobility.

Tui-na is an essential physical therapy method in Chinese traditional medicine (21, 22), with its origin dating back to the Shang Dynasty around 2,700 B.C. Over time, as society and economy developed and cultural exchanges deepened, Tui-na gradually evolved into a technique guided by the ethics of traditional Chinese medicine and modern scientific theories (23). Its technical treatment primarily focuses on relaxation-based manipulation, complemented by movement-based techniques. Following the principle of combining motion and static, tendon and bone, and gradual progression (24), Tui-na has proven to be highly effective in treating skeletal muscle diseases. In clinical practice, Tui-na practitioners employ various manipulative techniques to target specific areas of the body or acupuncture points, promoting optimal nerve and muscular tissue function, enhancing meridian and collateral circulation, relaxing soft tissues, restoring flexibility, and alleviating muscle spasms and pain symptoms (25, 26).

## Study design and participants

**Figure 1:**
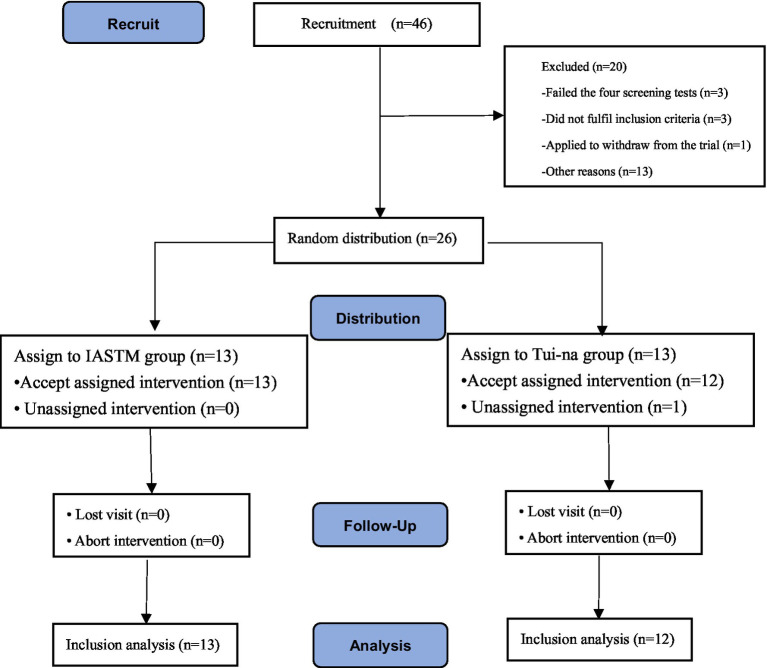
Flowchart of subject recruitment during the trial period.

The study is a comparative study with a randomized double-blind controlled intervention design where the intervention group underwent IASTM treatment, while the control group received Tui-na manipulation therapy for twice a week, with a 2–3 day interval between each session, for a total of 4 weeks (see Figure 1). Subjects were drawn from different sport-specific majors of the school, and physical therapy interventions were administered to the subjects by a national team sports rehabilitator who performed the manipulation (first author).

During and directly post intervention, the participants were assessed through a test battery consisting out of Lysholm knee score, VAS score, muscle strength, and modified Thomas test, while pain was assessed up to 6 months post intervention.

Students who met the inclusion and exclusion criteria were selected and at random appointed to either the intervention or control group. A total of 46 people were recruited for the trial, and according to the exclusion and inclusion criteria, 26 subjects with different specialities who met the criteria for PFPS were finally selected.

### Inclusion and exclusion criteria

The focus of the study was on four tests: patellar sliding trajectory, patellar passive sliding test, patellar lateral tilt assessment, and Waldron test (27, 28). These tests were used to determine the presence of patellofemoral pain or dysfunction. This intervention is a comparative study with a randomized double-blind controlled intervention design. The intervention will take place from September 10 to October 19, 2022 at the National Fitness Center and Sports Rehabilitation Center of Wuhan Sports Institute. Furthermore, this intervention has been registered on the ISRCTN platform (URL: www.isrctn.com; registration number: ISRCTN880989).

Inclusion criteria are as follows: ① age between 18 and 35 years old; ② ability to cooperate with rehabilitation physiotherapy, training, and follow-up, and willingness to participate in the study without withdrawing without reason; ③ patellofemoral joint pain lasting for more than 6 weeks; ④ occurrence of anterior knee pain during walking, running, jumping, going up and down stairs, and other physical activities; ⑤ knee pain during specific load exercises; and ⑥ positive results of the patellofemoral trajectory test after the four items.

Exclusion criteria are as follows: ① presence of external injuries to the knee joint, such as impact, blows, cuts, burns, contusions, etc., or presence of skin rupture bleeding or subcutaneous bleeding; ② presence of congenital abnormalities of the bone structure; ③ presence of inflammation, patellar subluxation or subluxation, and ligament injuries of the knee; ④ arthroscopy of the knee within the last year; ⑤ presence of injuries or discomforts to other parts of the body; and ⑥ participation in lower limb-related sport loading exercises during the test period.

### IASTM intervention group

The intervention group underwent intervention with instrumental therapy IASTM. At the onset of the intervention, the participants were instructed to receive treatment in various positions, which included the following steps: supine position with external rotation of the thigh and flexed knee, relaxation of the medial head of the quadriceps muscle; supine position with flexed and adducted knee, calf placed outside the bed, relaxation of the rectus femoris muscle, and intermediate femoris muscle. Supine position with knee flexed to neutralize the knee joint and loosen the soft tissues around the patella. Lateral position with knees bent, releasing the lateral head of the quadriceps and tensor fascia lata. Prone position with focus on releasing the hamstring muscle group, particularly the posterolateral biceps femoris. Please refer to Table 1 for specific instructions.

**Table 1:** Steps in IASTM treatment.

Type of tool	Workflow	Strength	Time	Purpose
C—Probe knife	Fascial lubricant is evenly applied to the treatment area, followed by controlled C-probe knife pressure applied in the direction of muscle fibers. The application can be from top to bottom or bottom to top at a 45° angle.	Low	1 min	The instrumental treatment rhythm was tailored to the subject’s needs, targeting areas of fascial densification or granulation (29–31).
A—Shark knife	The Shark Knife employs slow, repetitive pressure slides in areas of high resistance, such as fascial densification or trigger points (32).	Low-Middle	3–5 min	Soft tissues were loosened during both resting and maximal lower limb extension to restore elasticity, alleviate stiffness, and alleviate or eliminate pain points.
B—Bat knife	The Batblade applies pressure at a 45° angle, moving in both top-to-bottom and bottom-to-top directions with small, repetitive glides over areas of fascial densification or painful points.	Medium-High	3 min	Highly concentrated and intense pressure was applied for deep muscle release.
Big M knife	Subjects performed continuous knee flexion and extension or leg abduction and adduction movements with passive fascial knife pressure, synchronized with their respiration.	Medium-High	5–8 min	Dynamic release of deep muscle groups improved intermuscular glide and restored joint range of motion.
S-Hook knife	The Hook Knife targets localized stiffness and painful points, applying perpendicular sliding pressure to the muscle fibers’ direction.	Low-Middle	1–3 min	Deeper relaxation of excitation points.

### Tui-na manipulation control group

Tui-na manipulation therapy is a relaxation-based technique that incorporates both movement and static manipulation. It follows the principles of combining movement and static positions, as well as targeting tendons and bones, with a gradual progression (33). Various methods such as point-and-press, press-and-knead, gun, flexion-extension, and push were used to target specific acupuncture points around the knee joint. These points include the Blood Sea Point, Inner Knee Eye, Outer Knee Eye, Yangling Spring, Yinling Spring, Liangqiu Point, Zhizhong Point, and Zusanli Point, with the aim of promoting relaxation (34). The therapy focused on loosening the anterolateral, medial-lateral, posterior, and anterolateral thighs and calves. Each session of Tui-na manipulation therapy lasted for 30–45 min. Following the therapy, traditional lower limb strength training was performed for 15–20 min. The specific procedure is outlined in Table 2.

**Table 2:** Steps in Tui-na manipulation therapy.

Posturing	Method	Intensity	Time	Purpose
Lying flat with lower limbs in a relaxed position	The therapist applies significant pressure to the quadriceps muscle groups on the inner and outer sides of the thigh using the palm and forearm.	Low-Middle	1–2 min	Gradual adaptation to manual massage rhythm induces nerve relaxation and reduces stress response.
Lying flat with thighs in 90° knee flexion and abduction	In the static technique, the therapist uses the inner forearm for rolling pressure on the inner thigh, focusing on areas of stiffness and painful points. For the dynamic approach, a lubricant is applied, and the subject actively moves their leg inward, exerting pressure with both thumbs from knee to hip.	Medium-High	2–5 min	Subjects were screened to identify areas of fascial densification and tender points in the medial thigh, followed by rolling release to improve flexion and adduction range of motion.
Lying flat with lower legs hanging out naturally from the edge of the bed	In static treatment, root kneading pressure and forearm rolling pressure are applied to the quadriceps muscle in three different states: maximal flexion, maximal extension, and rest. In the dynamic method, as the subject performs knee flexion and extension, the therapist applies centripetal pressure with two fingers from the hip to the top of the knee joint and centrifugal pressure from the top of the knee joint to the hip.	Medium-High	2–5 min	Progressive muscle relaxation during centripetal and centrifugal movements of the quadriceps increases intermuscular glide and restores knee joint extension range of motion.
Lying on the side with the lower limbs in natural flexion	The iliotibial fascia and tensor fasciae latae muscle on the lateral thigh are treated with root kneading pressure and forearm rolling pressure in three positions: maximal thigh flexion, maximal extension, and rest in static treatment. During dynamic treatment, as the subject performs knee abduction and adduction movements, two fingers are used to apply pressure to the iliotibial bundle, moving from the hip to the posterior-lateral side of the knee for abduction and from the lateral side of the knee to the hip for adduction.	Medium-High	2–5 min	Gradual loosening of the outer thigh soft tissues facilitates increased intermuscular glide, aiding knee joint range restoration during abduction and adduction movements.
Lie prone	For the biceps femoris muscle on the posterior lateral thigh, static therapy involves root kneading pressure and forearm rolling pressure in three states: maximal flexion, maximal extension, and rest. In the dynamic approach, during knee flexion and extension, two fingers apply centripetal pressure from the hip toward the posterior upper part of the knee joint and centrifugal pressure from the posterior upper part of the knee joint toward the hip.	Medium-High	2–5 min	Manipulative massage targets the biceps femoris muscle on the lateral thigh, enhancing knee joint flexion ability.
Lying flat, sideways, and prone	Regarding the acupuncture points around the knee joint, they are treated through pressing, kneading, and the gun method under different postural conditions in static treatment. In the dynamic method, the acupuncture points are pressed and held while the subject actively performs knee joint movements, including flexion, extension, abduction, and adduction.	Medium-High	30–60 s per point	By applying more concentrated and intense pressure on acupuncture points and pain points to release deeper muscles, pain is reduced or eliminated, and knee range of motion is increased.

### Post intervention knee functionality assessment

#### Lysholm knee score

The Lysholm Knee Score (35) is a commonly used clinical tool to evaluate knee function and symptom severity. It was utilized to assess the knee function of patients before and after treatment. This scoring system evaluates eight aspects, including pain, instability, atresia, degree of swelling, lameness, stair climbing, kneeling, and brace use, with a total score of 100. Pain and instability criteria were each given 25 points. Scores below 65 were considered poor functional status, 65–83 as satisfactory, 84–94 as good, and 95–100 as excellent. Higher scores indicate better knee function, and therefore, the scores can be used to determine the effectiveness of the treatment.

#### VAS score

The Visual Analog Scale (VAS) (36) for Pain is a widely used tool for assessing pain. Its purpose is to measure an individual’s subjective perception of pain intensity. Typically, it is presented as a straight line or scale, with “no pain” and “most severe pain” labeled at the ends. Participants are instructed to mark the point on the line that corresponds to their perceived pain intensity. To evaluate the level of patellofemoral pain before and after treatment, the VAS for pain was employed. This scale ranges from 0 to 10 points, with higher scores indicating greater pain intensity. A score of 0 represents no pain, while a score of 10 represents unbearable pain.

### Lower limb extensor maximal isometric muscle strength test

Enhanced strength and neuromuscular control in the lower limbs can improve functional performance and reduce strain on the patellofemoral joint in patients with patellofemoral pain syndrome (PFPS) (37). Therefore, this intervention utilized a lower limb extensor maximal isometric muscle strength testing system (model: dr. wolff sports & prevention) before and after treatment. This testing system provides a reliable means for rehabilitation instructors to diagnose and assess the patient’s lower limb functional training and develop a rehabilitation program. During the testing process, the subject was positioned with the waist and hip close to the device, and both lower limbs were set at a fixed knee angle of 90° for the knee extension force test. In the muscle strength test, the peak force was recorded when the subject could no longer exert force, and three tests were conducted to obtain an average of the three peak strength values.

### Modified Thomas test

**Figure 2:**
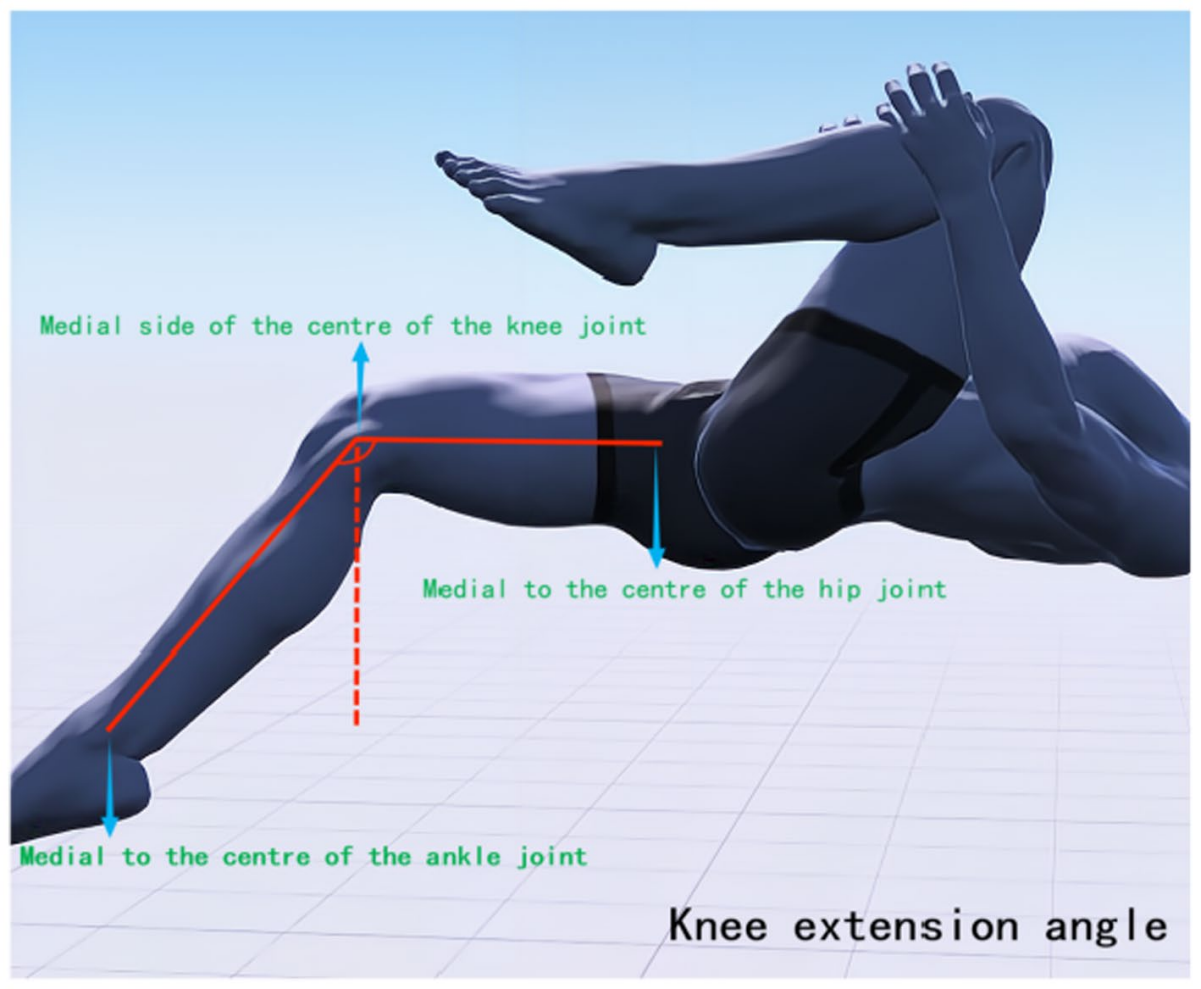
Knee extension angle.

**Figure 3:**
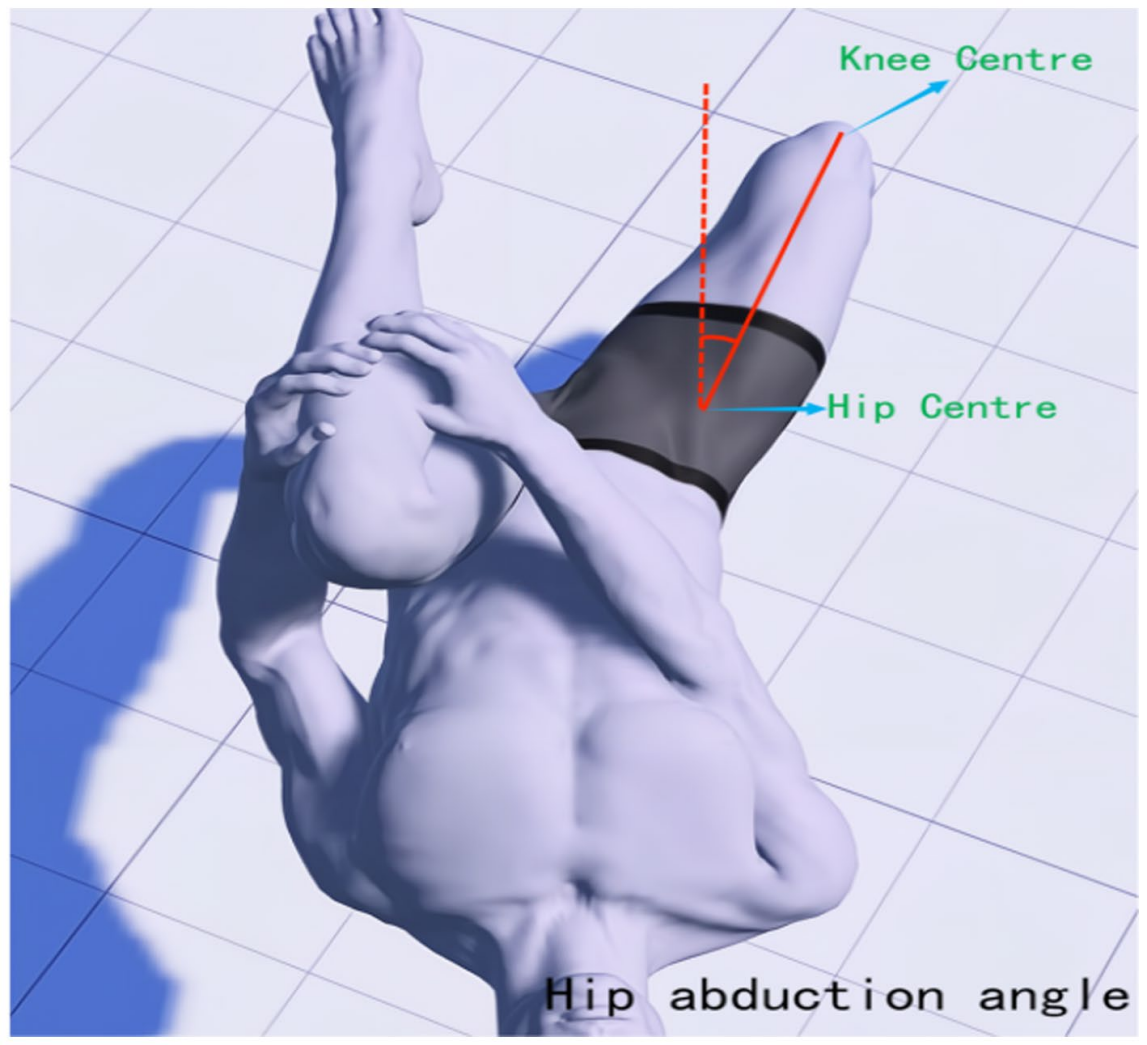
Hip abduction angle.

**Figure 4:**
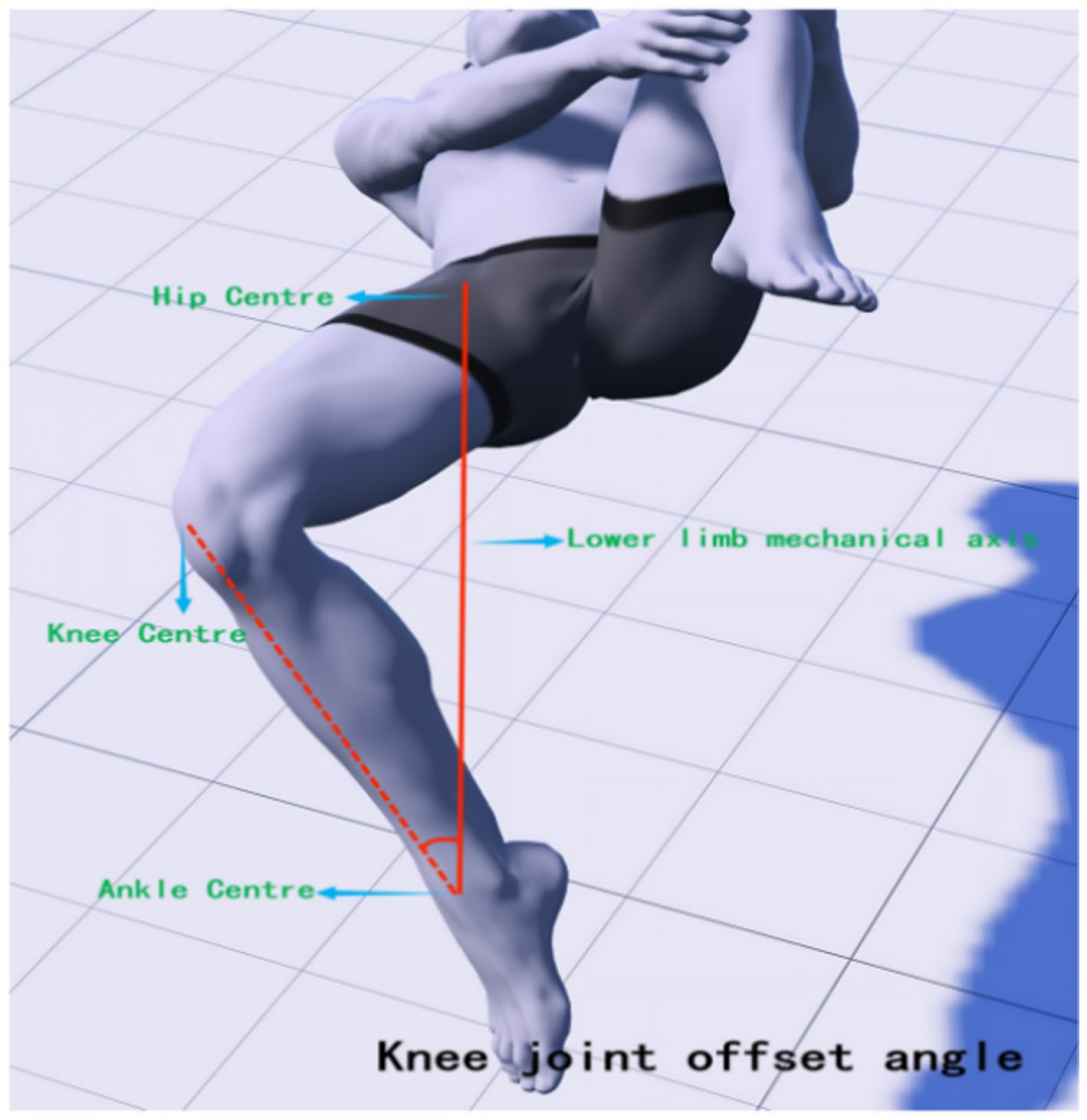
Knee joint offset angle.

The Modified Thomas Test (MTT) is a method used to assess the flexibility of the iliopsoas, rectus femoris, and vastus tensor muscles. It has been found to be highly reliable for testing muscle tone in the lower limbs (38, 39). Research suggests that an increased knee joint valgus angle may be a significant factor in knee joint injury and pain, with correlations found between reduced strength of hip abductors, external rotators, extensors, and knee flexors, and an increased knee joint valgus angle (40, 41). Therefore, it is possible to intervene through physical therapy to restore abnormal muscle tension around the knee joint, thereby reducing the risk of knee joint pain. In this study, high-precision joint mobility angle measuring tape was used to observe changes in lower limb joint angles before treatment, after the first treatment, and after 4 weeks of treatment. During the test, subjects were asked to lie down on the edge of the bed, with their hands holding the proximal knee end of the tibia of the healthy leg, and to observe whether the femur was parallel to the bed surface. If there was an angle between the femur and the bed, it indicated shortening tension of the iliopsoas muscle (hip flexor). The state of the affected leg was also observed. Excessive abduction of the hip joint indicated tension of the broad fascia tensor muscle, while excessive adduction indicated tension of the pectineus. If the leg presented the state of calf inwardly retracted femur outwardly (similar to the position of a shuttlecock), it indicated tension of the internal femoral rotator muscle. If the angle between the knee joint and the calf was too large, it indicated tension of the rectus femoris. During the tests, the interference of iliopsoas muscle tension was excluded. The mechanical axis position and the location of each joint center point of the hip-knee-ankle and lower limb hip-knee-ankle (HKA) were determined (42), with the line connecting the hip center (located deep in the central inguinal region, flush with the greater trochanter and the center of the femoral head) and the ankle center (the midpoint of the width of the talus) as the axis. The muscle groups affecting the knee joint status were mainly recorded, and MTT was performed again after IASTM treatment. The knee extension angle (measured at the medial knee joint center, see Figure 2), hip abduction angle (measured at the hip center, see Figure 3), and knee offset mechanical axis angle (measured at the ankle joint center against the hip center as an axis, see Figure 4) were measured.

### Statistical analysis

All statistical analyses of data from this intervention experiment were done independently by the first author. SPSS 26.0 statistical software was utilized in this study for data reading, testing, and statistical analysis. The subjects’ basic information was analyzed using an independent samples *t*-test. Measurement information was found to conform to the normal distribution, and the presence of three time-point measurement indicators was analyzed using repeated measures ANOVA. Paired *t*-tests were conducted to analyze the two time-point measures before and after treatment. A value of *p* of less than 0.05 was considered to indicate a statistically significant difference.

The sample size was calculated using G*Power 3.1.9.7 for analysis. Since there are repeated measures and interaction ANOVA between the different intervention groups, we selected “F tests, ANOVA: Repeated measures, between factors, Type of power analysis” and “*A priori*: Compute required sample size—given a, power, and effect size” for the software calculations. The specific parameters used were: “Effect size *f* = 0.25, α err prob. = 0.05, Power (1-B err prob) = 0.8, Number of groups = 2, Number of measurements = 3, Corr among rep measures = 0.” The final sample size result was calculated as 44/3 = 14.6 ≈ 15. The sample size of this intervention is 25 cases, which aligns with the result calculated by G*Power.

## Results

### Comparison of basic information on research subjects

A total of 46 people were recruited for the trial, and according to the exclusion and inclusion criteria, 38 subjects with different specialties who met the criteria for PFPS were finally selected. The patients were divided into the IASTM group (*n* = 13) and the nudging group (*n* = 12) using the random number table method (see Figure 4). There was no significant difference (*p* > 0.05) between the subjects in terms of gender, age, height, body mass, and duration of disease (see Table 3).

**Table 3:** Comparison of basic information of subjects in two groups.

Projects	IASTM group (*n* = 13)	Tui-na group (*n* = 12)	*t*	*p*
Gender (m/f, *n*)	8/5	6/6	1.915	0.08
Age (x̄, years)	21.67 ± 1.969	21.33 ± 2.462	0.456	0.10
Height (x̄, cm)	176.17 ± 3.950	171.00 ± 9.293	1.785	0.04
Body mass (x̄, kg)	71.58 ± 11.373	65.54 ± 15.63	0.734	0.48
Duration of pain (x̄, weeks)	20.792 ± 16.30	12.92 ± 8.34	1.740	0.12

### Lysholm knee score

The results of ANOVA by repeated measurements of Lysholm’s knee before treatment, after the first treatment, and at different time points for 4 weeks of treatment showed that there was no significant effect of group changes on Lysholm scores, *F* = 2.02, *p* = 0.17; there was a significant effect of measurements at different time points on Lysholm scores, *F* = 45.11, *p* < 0.001; however, there was no significant effect of group and time point had no significant effect on Lysholm score interaction, *F* = 68.56, *p* = 0.49.

When comparing between groups, there was no significant change in Lysholm score between the test and control groups until after 4 weeks of treatment (*p* = 0.005).

When comparing within groups, there was a significant change between the two treatments and pre-treatment after the first and after 4 weeks of treatment (*p* < 0.05). In contrast, there was no significant change in Lysholm scores between the two-time points after the first treatment and after 4 weeks of treatment (*p* > 0.05) not statistically significant. The statistical results are shown in Tables 4, 5.

**Table 4:** Results of two-factor repeated-measures ANOVA for changes in test metrics at three-time points on the Lysholm Rating Scale.

Lysholm repeated evaluation F-test			
	*F*	*p*	Bias η^2^
Group main effect	2.02	0.17	0.09
Point-in-time main effect	45.11	0.000	0.68
Group × time point	68.56	0.57	0.03

**Table 5:** Results of comparison of changes in test values and means at three-time points on the Lysholm Rating Scale.

	Before treatment	After the first treatment	After 4 weeks of treatment	Multiple are comparable
	M ± SD	M ± SD	M ± SD	
IASTM group (*n* = 13)	62.46 ± 4.17	90.82 ± 4.01^*&^	93.36 ± 2.93^*&^	Before treatment < after the first treatment < after 4 weeks of treatment
Tui-na group (*n* = 12)	61.33 ± 3.99	84.25 ± 3.84^*&^	85.83 ± 2.80^*&^	Before treatment < after the first treatment < after 4 weeks of treatment

^*^represents a significant difference in the change in Lysholm score before treatment compared to before treatment when comparing within groups (*p* < 0.001); ^&^represents no significant change when comparing different time points within the two groups (*p* > 0.05); M ± SD indicates mean ± standard deviation.

### VAS scale scores

The results of repeated measures ANOVA of the VAS scale at different time points before treatment, after the first treatment, and at 4 weeks of treatment showed that the change in group did not have a significant effect on the VAS scores, *F* = 2.99, *p* = 0.10; there was a significant effect of measurements at different time points on the VAS scores, *F* = 3,129, *p* < 0.001; and there was no significant interaction between number of measurements and group for the VAS scores effect, *F* = 0.65, *p* = 0.47.

When comparing groups, there was no significant change in VAS scores measured at the three further time points between the test and control groups (*p* > 0.05).

When comparing within groups, there was a significant change between two treatments after the first treatment vs. 4 weeks of treatment and pre-treatment (*p* < 0.05). Within the test group, there was no significant change in VAS scores immediately after the first treatment vs. after 4 weeks of treatment (*p* > 0.05), which was not statistically significant. The statistical results are shown in Tables 6, 7.

**Table 6:** Results of two-factor repeated-measures ANOVA for changes in test metrics at three-time points on the VAS scale.

VAS repeated evaluation F-test			
	*F*	*p*	Bias η^2^
Group main effect	2.99	0.10	0.12
Point-in-time main effect	31.29	0.000	0.59
Group × time point	0.65	0.47	0.03

**Table 7:** Results of comparison of changes in test values and means at three-time points on the VAS scale.

	Before treatment	After the first treatment	After 4 weeks of treatment	Multiple are comparable
	M ± SD	M ± SD	M ± SD	
IASTM group (*n* = 13)	14.92 ± 2.86	4.92 ± 1.64^*&^	2.25 ± 1.38^*&^	Before treatment > after the first treatment > after 4 weeks of treatment
Tui-na group (*n* = 12)	20.58 ± 2.86	7.42 ± 1.64^*^	3.58 ± 1.38^*^	Before treatment > after the first treatment > after 4 weeks of treatment

^*^ represents a significant difference in the change in VAS scores compared to pre-treatment (*p* < 0.05); ^&^represents no significant change in VAS scores after the first treatment in the group compared to after 4 weeks of treatment (*p* > 0.05); M ± SD indicates mean ± standard deviation.

### Maximum strength of lower extremity extensor muscles

The maximum extensor strength of the lower limbs was only tested before treatment and after 4 weeks of treatment and compared within the group; the extensor strength of the lower limbs of the control group and the intervention group before and after treatment were improved, and the difference was significant (*p* < 0.001). Comparison between groups; test strength values did not change significantly (*p* > 0.05) and were not statistically significant. See Table 8.

**Table 8:** Comparison of lower limb strength data between the two groups before and after treatment.

Groups	*n*	Pre-treatment	After 4 weeks of treatment	*t*	*p*
Tui-na group	12	90.97 ± 61.55	109.07 ± 57.97	−6.837	<0.001
IASTM group	13	76.15 ± 39.25	92.52 ± 42.01	−4.984	<0.001
*t*		−0.675	−0.758		
*p*		0.514	0.464		

### Modified Thomas test

ANOVA of repeated measurements of the MTT test at different time points before treatment, after the first treatment, and at 4 weeks of treatment showed that there was a non-significant effect of both the groups of hip abduction angle and knee offset angle on the angular change, (*F* = 0.59, *F* = 45), (*p* = 0.45, *p* = 0.51); there was a significant effect of the group of knee extension angle on the angular change (*F* = 5.38, *p* < 0.05), and there was a significant effect of the number of measurements on angular change (*F* = 188.17, *F* = 118.84, *F* = 36.38), (*p* < 0.001); the interaction between group and number of measurements on angular change for the three joint angle measurements was not significant, (*F* = 1.60, *F* = 0.71, *F* = 0.88), (*p* = 0.22, *p* = 0.53, *p* = 0.38).

When comparing between the groups, there was a significant change in the knee extension angle measurements between the test and control groups only before and after the first treatment (*p* < 0.05), and no significant change in the values of the three angle measurements at other time points (*p* > 0.05).

When comparing within groups, the three angle measurements immediately after the first treatment and at two time points after 4 weeks of treatment significantly differed from the pre-treatment measurements (*p* < 0.05). Whereas only the hip abduction angle measurements changed significantly (*p* < 0.05) within the test group at both time points immediately after the first treatment vs. 4 weeks post-treatment, the knee extension angle and hip abduction angle measurements changed significantly (*p* < 0.05) within the control group. The statistical results are shown in Tables 9, 10.

**Table 9:** Results of two-factor repeated-measures ANOVA for changes in joint angles for MTT testing.

	Knee extension angle			Hip abduction angle			Knee joint offset angle		
	*F*	*p*	Bias η^2^	*F*	*p*	Bias η^2^	*F*	*p*	Bias η^2^
Group main effect	5.38	0.03	0.196	0.59	0.45	0.026	0.45	0.51	0.02
Point-in-time main effect	188.17	0.000	0.90	118.84	0.000	0.84	36.38	0.000	0.63
Group × time point	1.60	0.22	0.07	0.71	0.53	0.03	0.88	0.38	0.04

**Table 10:** Results of comparison of numerical changes and mean values of MTT test joint angles.

Group	Projects	Before treatment	After the first treatment	After the final treatment	Multiple are comparable
		M ± SD	M ± SD	M ± SD	
IASTM group (*n* = 13)	Knee extension	121.85 ± 1.75^#^	101.82 ± 1.57^*#^	97.16 ± 1.72^*^	Before treatment > after the first treatment > after 4 weeks of treatment
	Hip abduction	39.92 ± 3.79	14.37 ± 1.522^*a^	8.30 ± 1.10^*a^	Before treatment > after the first treatment > after 4 weeks of treatment
	Knee Offset	13.93 ± 2.47	7.00 ± 0.98^*^	5.40 ± 0.90^*^	Before treatment > after the first treatment > after 4 weeks of treatment
Tui-na group (*n* = 12)	Knee extension	127.56 ± 1.75^#^	106.86 ± 1.57^*#a^	98.06 ± 1.72^*a^	Before treatment > after the first treatment > after 4 weeks of treatment
	Hip abduction	34,026 ± 3.19	14.05 ± 1.52^*a^	7.28 ± 1.10^*a^	Before treatment > after the first treatment > after 4 weeks of treatment
	Knee offset	17.07 ± 2.58	7.2 ± 1.02^*^	5.74 ± 0.94^*^	Before treatment > after the first treatment > after 4 weeks of treatment

^*^represents a significant difference in angular change from pre-treatment in within-group comparisons (*p* < 0.05); ^a^ represents a significant difference in angular change after the first treatment and after 4 weeks of treatment in within-group comparisons (*p* < 0.05); ^#^ represents a significant difference in angular change between the two intervention groups (*p* < 0.05); M ± SD indicates mean ± standard deviation.

### Long-term follow-up results

The subjects were followed up at three time points: 1, 3, and 6 months after the end of the trial. The follow-up included monitoring the development and frequency of pain. The results showed that most people did not experience significant knee pain on a regular basis at the six-month mark.

In the IASTM group, only one male subject occasionally experienced pain when under load 1 month after the intervention. Additionally, one male and two female subjects experienced occasional soreness and weakness while exercising. In the nudging group, one female subject experienced occasional pain while bearing a load, and one male subject experienced occasional pain while walking up and down stairs.

During the first 3 months after the intervention, two female subjects in the IASTM group experienced occasional pain while exercising up and down stairs and with a load, respectively. In the push-up group, one male subject developed occasional pain while training to bear the load, and two female subjects experienced occasional pain while walking up and down stairs.

Within 6 months after the intervention, one male and one female subject in the IASTM group developed occasional pain while exercising up and down stairs, and one female subject experienced occasional pain while performing weight-bearing exercises. In the nudging group, two female subjects developed occasional pain during exercise with load bearing, and two female subjects and one male subject developed occasional pain during exercise up and down stairs.

In summary, during the first 6 months, only a few individuals experienced occasional discomfort or pain in a specific area during exercise, and most of the pain was only present during weight-bearing exercises. No significant pain was reported during daily activities such as lying, sitting, standing, and walking. The joint group had no subjects experiencing pain in the short-term period of 1 month, and the fewest number of subjects reported pain or discomfort over the 6-month period. Both intervention groups showed positive effects on the symptoms of PFPS patients in the initial and middle stages of treatment, with the combined group demonstrating a better sustained therapeutic effect compared to the other two groups. The specific results can be seen in Tables 11, 12.

**Table 11:** Follow-up results for pain-producing states.

State of affairs	IG/1 M	TG/1 M	IG/3 M	TG/3 M	IG/6 M	TG/6 M
Lie flat	-	-	-	-	-	-
Walking	-	-	-	-	-	-
Sitting	-	-	-	-	-	-
Standing	-	-	-	-	-	-
Carrying loads	m = 1	f = 1	f = 1	m = 1	f = 1	f = 2
Walking up and down stairs	-	m = 1	-	f = 2	m = 1, f = 1	m = 1, f = 2
Soreness and weakness	m = 1, f = 2	-	f = 1	-	-	-

TG, Tui-na group; IG, IASTM group; m, Male; f, Female; M, Month; “-,” no observation.

**Table 12:** Follow-up results on frequency of pain generation.

Frequency	IG/1 M	TG/1 M	IG/3 M	TG/3 M	IG/6 M	TG/6 M
Occasional	m = 1	m = 2, *f* = 1	f = 2	m = 1, f = 2	m = 1, f = 2	m = 1, *f* = 4
Frequent	-	-	-	-	-	-
Pain-free	m = 4, *f* = 8	m = 4, f = 5	m = 5, f = 6	m = 5, f = 4	m = 3, *f* = 6	m = 5, f = 3

TG, Tui-na group; IG, IASTM group; m, Male; f, Female; M, Month; “-,” no observation.

## Discussion

Instrument-assisted soft tissue mobilization therapy and Tai-na massage manipulation techniques are commonly used in physiotherapy for musculoskeletal pain rehabilitation. Both treatments have their own characteristics and advantages, particularly IASTM treatment, which utilizes tools to target deeper myofascial layers and provide longer-lasting therapeutic effects (43). Therefore, this study aimed to compare the effects of instrumental treatment—IASTM and Tui-na manipulation therapy—on patients with lateral epicondylitis of the humerus. The results indicated that both IASTM and Tui-na manipulation treatments significantly reduced pain, improved knee flexibility, and increased joint range of motion in PFPS. However, the immediate effect of IASTM treatment was more pronounced, and the improvement in pain after 4 weeks of treatment was superior to that of the Tui-na group. Follow-up results at 4–12 weeks after treatment also demonstrated that IASTM had better efficacy in the short to medium term for lateral epicondylitis of the humerus.

The therapeutic tools utilized in IASTM therapy are specifically designed to aid in the treatment of soft tissue injuries and facilitate the recovery and healing of damaged soft tissues (44, 45). Typically performed by a specialized clinician or therapist, this therapy targets the body’s soft tissues to address issues such as lack of elasticity, hypotonia, and muscle tension and stiffness (46). IASTM involves the use of tools, such as a handheld acupressure massager, as an intermediary medium to be applied when examining an area that exhibits muscle laxity. By altering the fascial structure and restoring it to its natural proportions through stereo modeling, the therapy aims to restore the body’s functional level (43). During IASTM treatment, the therapist employs tools and instruments to apply pressure and eddy currents to the subcutaneous tissues, stimulating the metabolic vitality of the tissues and aiding in post-traumatic tissue healing. This therapy also promotes lymphatic circulation and accelerates the transportation of oxidative substances (47). As an extracorporeal physical intervention therapy, IASTM is non-invasive and free of side effects, making it a suitable adjunctive therapy for most musculoskeletal pain. These characteristics bear similarities to massage manipulation therapy.

Tui-na is a therapy in Chinese medicine that aims to promote the flow of meridians, qi, and blood, as well as regulate the function of internal organs. It utilizes techniques such as grasping, kneading, pointing, and squeezing to eliminate fatigue and relieve nervous tension (48, 49). Tui-na can effectively treat various conditions including neck, shoulder, lower back, and leg pain, headaches, insomnia, and indigestion. When combined with other traditional Chinese medicine (TCM) therapies like acupuncture, moxibustion, and herbal medicine, Tui-na can provide even greater therapeutic benefits (50–52). Unlike invasive treatments such as drug injections or surgeries, Tui-na is a non-invasive approach with minimal risks and side effects. Additionally, Tui-na can tailor treatment plans to suit individual patients’ specific conditions, ensuring personalized care. Compared to other treatment methods, Tui-na requires simpler equipment and is relatively cost-effective. However, as a manual therapy that heavily relies on hand manipulation to treat injuries, Tui-na therapists are at a higher risk of developing musculoskeletal disorders due to repetitive strain (53–55)and poor posture (56, 57). In this study, both IASTM and Tui-na manipulation effectively improved elbow pain and function immediately after the first treatment in both intervention groups. Notably, subjects who received a single session of IASTM treatment reported a significant “feeling of lightness” on the affected side and were able to perform various functional movements of the elbow, indicating a significant immediate effect. After 4 weeks of treatment, IASTM treatment showed significant improvements in pain, function, and range of motion compared to pre-treatment and initial treatment. During the follow-up period from 4 to 16 weeks after treatment, IASTM treatment demonstrated superior results in terms of pain relief and functional improvement compared to push therapy, indicating the short to medium-term efficacy of IASTM treatment.

Patients with patellofemoral pain syndrome (PFPS) often experience pain that significantly impacts their quality of life. This pain is particularly felt when the elbow is extended or compressed, resulting in drawing or pressing pain. The most notable pain points in these cases are known as myofascial trigger points (MTrPs) (32). Myofascial trigger points are hyper-excitable points found within areas of tight skeletal muscle or fascia. However, it has been recognized that deep fascia, including tendons and extra myocardial fascia, may be a potential source of pain (30, 58, 59), rather than the skeletal muscle itself. This is because fascia is densely innervated with nerve endings, making it more likely to transmit pain signals and cause significant discomfort for the patient (60, 61). Myofascial trigger points are typically found in areas of fascial densification, which has been linked to changes in the viscosity of loose connective tissue caused by factors such as diet, exercise, and overuse. The higher the viscosity of the loose connective tissue and the thicker the fascia, the more pronounced the fascial densification becomes. However, it is important to note that fascial densification is reversible and can be improved through physiotherapeutic interventions aimed at reducing fascial thickness and restoring the elasticity of the loose connective tissue, ultimately improving deep fascial densification (58, 59). In this context, the mediators of loose connective tissue that facilitate sliding between adjacent layers of fascia, particularly hyaluronic acid (H.A.) (62), play a critical role in assisting movement between muscle fibers. When the body experiences long-term chronic or overuse problems, the viscosity of hyaluronic acid increases, reducing the sliding between the fascia and making the fascial structure harder, resulting in fascial densification or fibrosis. During the sliding of fascial layers, nerve endings sense the body’s movement. Therefore, reduced sliding between fascial layers changes afferent nerve signals, triggering motor dysfunction and resulting in local discomfort or pain (63). In this intervention, IASTM treatment targets the densified area of the fascia around the patient’s knee joint. The intervention involves applying pressure to the densified area with two combined “chisel-like” forces: pressure that penetrates the dermis and subcutaneous tissue to reach the dense or granular areas and friction in a tangential direction. Since pressure alone cannot change the denseness of the fascia, sliding pressure on the soft tissue is necessary to achieve specific results. Therefore, when using a fascial knife, the therapist chooses an entry angle of about 45°, allowing the two forces to work together to solve the densification problem. The frictional force exerted on the rough tissue during fascial densification treatments causes vibrations of the molecules, and the kinetic energy of such movement is transferred between neighboring molecules, resulting in heat generation (64).

Therefore, an increase in temperature can transform the densified state of hyaluronic acid into a more mobile state, and localized heat production in the fascial tissue is more likely to change the densified area from a gel state to a sol–gel state. However, at the beginning of the treatment, the area of fascial adhesion may resist the release operation and cause acute discomfort. This phenomenon will persist, and the resistance and discomfort will gradually decrease as the friction generates sufficient heat to alter densification and convert the gel into a lysate. This is used to assess the strength and effectiveness of the treatment process. Studies have demonstrated that IASTM treatments can significantly elevate muscle and skin temperature and maintain it for over an hour (65). This external pressure stimulation enhances skin blood flow and accelerates local metabolism, providing fascial space for muscle engorgement and tension expansion, creating conditions for muscle strength training. Therefore, the use of instrumental treatment—IASTM is a favorable option for treating PFPS.

## Conclusion

In this study, both IASTM and Tui-na techniques have shown significant benefits in the treatment of PFPS. These interventions have effectively reduced pain, improved elbow flexibility, and increased joint range of motion in patients with PFPS. Particularly, IASTM treatment has demonstrated immediate and sustained effects in pain improvement. From the therapist’s perspective, using tools for treatment is easier and more convenient compared to unarmed treatment, as it does not strain or discomfort the hand (66). However, from the patient’s perspective, unarmed therapy is more acceptable. During unarmed therapy, patients experience softer and more relaxed sensations, with less raw pain and stress. The intensity of treatment increases gradually, which may be attributed to the therapist’s proficiency and technique. Physical contact allows for better management of strength and intensity. Combining these two treatment modalities may yield better results, with the most appropriate modality used at different stages of treatment. However, due to the small sample size and short duration of the study, the quantitative indexes used need to be more comprehensive and objective, and long-term follow-up is necessary. Additionally, the study population mainly consisted of athletes or physical education students, limiting the generalizability of the findings. Future studies should aim to expand the sample size to include various populations, utilize more objective indicators, and conduct randomized controlled trials comparing different physiotherapy treatments.

## Data availability statement

The original contributions presented in the study are included in the article/supplementary material, further inquiries can be directed to the corresponding author.

## Ethics statement

The studies involving humans were approved by Medical Ethics Committee of Wuhan Institute of Sports Ethical Review Form for Human Experiments. The studies were conducted in accordance with the local legislation and institutional requirements. The participants provided their written informed consent to participate in this study. Written informed consent was obtained from the individual(s) for the publication of any potentially identifiable images or data included in this article.

## Author contributions

YL: Conceptualization, Data curation, Formal analysis, Investigation, Methodology, Software, Supervision, Writing – original draft, Writing – review & editing. YW: Software, Validation, Formal analysis, Investigation, Resources, Writing – review & editing, Visualization, Supervision.
